# A multi-time-point modality-agnostic patch-based method for lesion filling in multiple sclerosis

**DOI:** 10.1016/j.neuroimage.2016.06.053

**Published:** 2016-10-01

**Authors:** Ferran Prados, Manuel Jorge Cardoso, Baris Kanber, Olga Ciccarelli, Raju Kapoor, Claudia A.M. Gandini Wheeler-Kingshott, Sebastien Ourselin

**Affiliations:** a Translational Imaging Group, Centre for Medical Image Computing (CMIC), Department of Medical Physics and Bioengineering, University College London, Malet Place Engineering Building, Gower Street, London, WC1E 6BT, UK; b NMR Research Unit, Queen Square MS Centre, Department of Neuroinflammation, UCL Institute of Neurology, 1st Floor, Russell Square House, 10-12 Russell Square, London WC1B 5EH, UK; c Department of Neuroinflammation, National Hospital for Neurology and Neurosurgery, Queen Square, London WC1N 3BG, UK; d Brain MRI 3T Center, C. Mondino National Neurological Institute, Pavia, Italy; e Dementia Research Centre, Department of Neurodegenerative Disease, UCL Institute of Neurology, Queen Square, London WC1N 3BG, UK

**Keywords:** Error correction, Multiple sclerosis, Lesions, Segmentation errors, Artefacts, MRI

## Abstract

Multiple sclerosis lesions influence the process of image analysis, leading to tissue segmentation problems and biased morphometric estimates. Existing techniques try to reduce this bias by filling all lesions as normal-appearing white matter on T1-weighted images, considering each time-point separately. However, due to lesion segmentation errors and the presence of structures adjacent to the lesions, such as the ventricles and deep grey matter nuclei, filling all lesions with white matter-like intensities introduces errors and artefacts. In this paper, we present a novel lesion filling strategy inspired by in-painting techniques used in computer graphics applications for image completion. The proposed technique uses a five-dimensional (5D), patch-based (multi-modality and multi-time-point), Non-Local Means algorithm that fills lesions with the most plausible texture. We demonstrate that this strategy introduces less bias, fewer artefacts and spurious edges than the current, publicly available techniques. The proposed method is modality-agnostic and can be applied to multiple time-points simultaneously. In addition, it preserves anatomical structures and signal-to-noise characteristics even when the lesions are neighbouring grey matter or cerebrospinal fluid, and avoids excess of blurring or rasterisation due to the choice of the segmentation plane, shape of the lesions, and their size and/or location.

## Highlights

• A multi-modality and multi-time-point lesion filling technique
• Lesions are filled with the most plausible texture rather than white matter.
• It preserves anatomical structures and signal-to-noise characteristics.

## Graphical abstract

**Figure:**
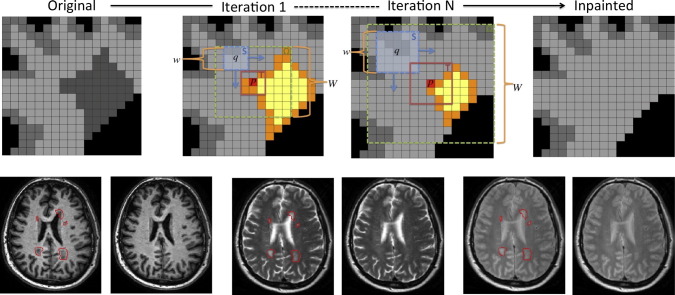

## Introduction

Multiple sclerosis (MS) is an immune-mediated demyelinating disease that affects both white matter (WM) and grey matter (GM). It is characterised pathologically by areas of inflammation, demyelination, axonal loss and gliosis scattered throughout the central nervous system. These pathological processes affect several quantitative MRI indices, and therefore can be indirectly measured with advanced imaging methods. Among these, tissue volume and, in particular, brain/tissue-specific atrophy, are very sensitive to subtle changes over a scale of a few months, making *in vivo* MRI measurements of these indices very appealing for studying the mechanisms of disease and for clinical trials. White matter plaques are relatively easy to detect using conventional MRI techniques, whereas grey matter lesions can be observed using specialised sequences, such as double inversion recovery (DIR) (Geurts et al., 2012) or phase sensitive inversion recovery (PSIR) (Sethi et al., 2012). MS plaques appear as areas of low-signal intensity and high-signal intensity compared with normal-appearing white matter (NAWM) on T1-weighted and T2-weighted sequences respectively. On the other hand, active lesions exhibit hyper-intense signals on gadolinium-enhanced scans (Lladó et al., 2012). Lesions and atrophy are two interconnected aspects of the disease, linked to different disease mechanisms, and both are extremely important for MS studies.

From an image processing perspective, MS lesions influence tissue segmentation, resulting in the misclassification of the GM and the WM. It has been suggested that MS lesions may affect the estimation of segmentation parameters, resulting in a shift of tissue boundaries (Chard et al., 2010), thus influencing the subsequent morphometric studies, including atrophy measurements. Thus, there is a clear need to reduce the negative impact that MS lesions may have on image analysis in order to improve tissue segmentation and longitudinal registration, increasing sensitivity to subtle changes, reducing the time-intervals and sample sizes needed for longitudinal studies and treatment trials.

Various techniques have been developed in recent years based on the concept of in-painting T1-weighted MRI images: Sdika and Pelletier (2008), Chard et al. (2010), Battaglini et al. (2012), Magon et al. (2014), Valverde et al. (2014), and Guizard et al. (2015).

In short, the process of lesion in-painting is based on filling a WM lesion with synthetic estimates of WM-like image intensities. The process of WM lesion in-painting is expected to reduce the overall algorithmic bias. Sdika and Pelletier (2008) presented three different in-painting algorithms. The first, denoted *basic in-painting* and inspired by Telea (2004), consists of filling the lesion ROI in an inner-radial manner using a Gaussian kernel average 3 × 3 × 3 of the neighbouring intensities. The second, called *local white matter in-painting* (LWMI), uses *a priori* information obtained from an image segmentation technique to iteratively fill the border of the lesions using a Gaussian kernel. Finally, the *global white matter in-painting* (GWMI) method fills the MS lesions with the mean intensity of the normal WM over the whole brain, meaning that all lesions will have the same intensity regardless of their neighbourhood.

Recently, Chard et al. (2010) developed the LEAP (LEsion Automated Preprocessing) technique with the aim of filling lesions as normal WM, reproducing the WM noise characteristics and avoiding operator intervention. This method starts by skull stripping the brain and applying a non-uniformity intensity correction algorithm. The normal tissue intensity distribution is modelled numerically as the sum of four Gaussian components representing GM, WM, CSF, and partial-volume voxels. Finally, the lesion ROI is filled with random samples from a Gaussian distribution with mean equal to the most probable WM intensity and a standard deviation equal to the WM full-width half maximum noise characteristics. This method is available at: http://www.nmrgroup.ion.ucl.ac.uk/. Valverde et al. (2014) and Magon et al. (2014) have then introduced variations to the LEAP method. Valverde et al. (2014) suggested to fill the volume in a slice-wise manner, whilst Magon et al. (2014) filled the lesions using the mean intensity of two voxel expanded neighbouring over the normal-appearing WM.

Similarly, Battaglini et al. (2012) presented a method based on replacing the lesion voxel intensities with values that are randomly sampled from an intensity distribution that is measured from the surrounding WM and GM voxels. The surrounding normal-appearing tissue volume is taken as the extra volume obtained by dilating the lesion ROI twice. Lesions are then filled with samples taken from the neighbouring histogram using a uniform random value passed through an interpolated version of the empirical cumulative distribution function of the neighbouring histogram. Both GM and WM voxels are included in the neighbouring histogram in order to represent the surrounding tissue and allow the filled lesions to best visually blend into its environment. This method is available as part of FSL (Jenkinson et al., 2012) at http://fsl.fmrib.ox.ac.uk.

Regardless of their approach, all these algorithms have been restricted to images of a specific MRI modality (*e.g.* T1-weighted scans), and require accurate lesion segmentation, especially when lesions are periventricular or when the methods are based on filling the lesions with values from the surrounding areas. They can also create shape gradients around the lesion ROIs, and are prone to errors coming from estimating WM distribution properties.

More recently, Guizard et al., 2013, Guizard et al., 2015 calculated the most similar patches using only the surrounding regions after pre-filling the lesions with the median of the image intensities of the surrounding of healthy tissues (Guizard et al., 2013). The same authors later introduced an hierarchical, concentric filling strategy, where distances between patches are computed over the full patch, the filling process is repeated with different weighting values and at multiple increasing resolutions (Guizard et al., 2015).

In the field of computer graphics, structurally aware in-painting algorithms used for scratch/object/text removal and photo restoration are common, with many of these algorithms permitting a user to simply erase an unwanted portion of an image without any prior knowledge about its composition. These techniques attempt to fill regions by synthesising plausible texture matches from the remainder of the image (Criminisi et al., 2003, Komodakis and Tziritas, 2007, Barnes et al., 2010). In doing so these algorithms are completely agnostic to the structure of the input image.

The most successful techniques for in-painting in computer graphics, here denoted as exemplar-based methods, attempt to fill the unknown ROI by simply copying content from the observed part of the images (Komodakis and Tziritas, 2007), under some constraints. This class of methods commonly divides the image into a large number of sub-images, or patches, followed by either a patch-search method Criminisi et al. (2003), or the use of the Non-Local Means algorithm (Buades et al., 2005). Finally, the intensities can be synthesised using either pixel values or patch-based textures from the most similar patch.

In this work, we formulate a multi-time-point, task-specific, patch-search algorithm for the purpose of filling MS lesions. This novel work offers three main advantages: first, due to its general formulation, the proposed algorithm should in theory be able to inpaint most types of MR images with repetitive patterns. Secondly, due to its contextual nature, the proposed algorithm is also more robust to over-segmentation of the lesion ROIs, thus reducing accuracy requirements when manually or automatically defining the in-painting region of interest. Thirdly, and finally, it allows filling lesions at different time-points (as in longitudinal studies) at once taking advantage of the information of the lesion evolution.

## Material and methods

The proposed lesion filling technique can be described in three main steps: (1) determining the patch with the most similar neighbourhood structure, (2) synthesising the intensity pattern from the best patch, followed by (3) a buffing step through the application of a minimal kernel-based convolution over the filled region.

First, we assume that we have a greyscale-valued 5D volume *I*^⁎^, composed of *n* different modalities or MRI sequences, over *t* time points, with each individual 3D volume being of size *X* × *Y* × *Z*. Each time point and modality has an associated lesion mask, here denoted as L.

Let the filled image *I* be defined as *I*(*p*) = *I*^⁎^(*p*) ∀* p* ∉ L, and as $I\left(p\right)=F\left(p\right)$ ∀p∈L , where *p* denotes the voxel location (*x*, *y*, *z*, *n*, *t*) in the image *I* and $F\left(p\right)$ is the function that synthesises the intensity of voxel *p*. We define Ω as a search region of size *W*^3^ voxels around voxel *p* (where *W* denotes the spatial search region size in voxels in each spatial direction). Within the region Ω, we define a 5D target patch *T*(*p*) of size *ntw*^3^ voxels (where w denotes the patch size in voxels), centred at a voxel *p*, and a search patch *S*(*q*) of size *ntw*^3^ voxels, centred in *q*, with *q* ∈ Ω and *q* ∉ L.

Given *w* and *W*, we propose to replace (or fill) the voxel intensity *I*^⁎^(*p*) with the intensity *I*^⁎^(*q*) if *S*(*q*) is the most similar patch to *T*(*p*), under the constraint that *q* is within the search region Ω, outside the lesion region L and that *q* ≠ *p*. Formally, a temporary estimate $\hat{I}\left(p\right)$ for all p∈L can be generated by finding $\hat{I}\left(p\right)=I\left(\hat{q}\right)$ with:

(1) $$\hat{q}=\underset{\forall q\in \Omega |\left(q\neq p\right)\wedge \left(q\notin L\right)}{\text{argmin}}D\left(T\left(p\right),S\left(q\right)\right)$$

where the distance D between two patches *T* and *S* is equal to

(2) $$D\left(T\left(p\right),S\left(q\right)\right)=\frac{\sum_{\forall i\in \left\{\mathrm{nt}w^{3}\right\}\left|\{T\left(p+i\right),S\left(q+i\right)\}\notin L\right)}{\left(I\left(p+i\right)-I\left(q+i\right)\right)}^{2}}{\kappa^{c}}$$

**Fig. 1:**
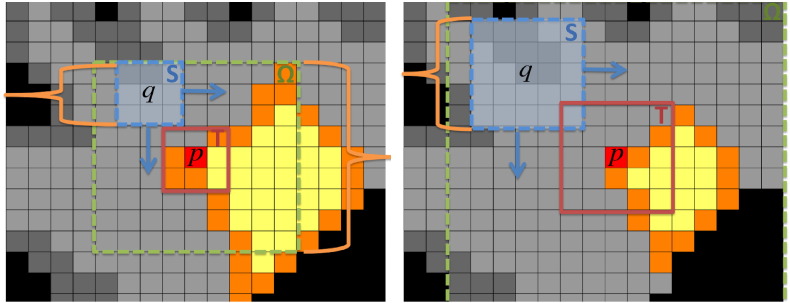
Algorithm scheme that illustrates two different iterations, with the voxels in orange denoting the ones respecting the hard constraint *κ* > *αntw*^3^. In short, the proposed method moves the search patch *S*, centred at *q*, within Ω, until it finds the location $\hat{q}$ where $S\left(\hat{q}\right)$ and *T*(*p*) are the most similar.

Here, *κ* is the cardinality of the set *i* ∈ {*ntw*^3^}|{*T*(*p* + *i*), *S*(*q* + *i*)} ∉ L, *i.e.* the number of voxels within the patches *T*(*p*) and *S*(*q*) that are not in the lesion region. Note that when *c* > 1, the denominator *κ*^*c*^ favours patches with more information. A further hard constraint can be added by defining *α* as the minimum required percentage of non-lesion voxels in a patch. This hard constraint can be formally defined as *κ* > *αntw*^3^, *i.e.* the cardinality of the set ∀* i* ∈ {*ntw*^3^}|{*T*(*p* + *i*), *S*(*q* + *i*)} ∉ L has to be more than *α*% of the patch size. If this constraint is satisfied, then *p* is marked as solve and is removed from the set L, otherwise, *p* remains in L. This process is repeated until L=∅, *i.e.*, every voxel *p* initially in L has an estimate $\hat{I}\left(p\right)$. The values of *p* are accessed in an inwards manner dependent on the distance between *p* and the boundary of the lesion, meaning that the lesion is inpainted radially in an inwards direction as depicted in Fig. 1.

Finally, when L=∅, $F\left(p\right)$ can then be estimated by buffing the estimates of $\hat{I}\left(p\right)$ using a convolution operation $F=C*\hat{I}$, where C is a minimal 6-neighbourhood clique (cross-shape) kernel with its centre voxel set to 1 and all other voxels set to K. The kernel C is then normalised so that the kernel density sums to 1. This buffing step increases the smoothness of the inpainted region, thus removing of spurious edges that might affect further analysis pipelines, *e.g.* atrophy estimation.

The presented algorithm has been included as standalone tool in the software package NiftySeg (niftyseg.sf.net) and as web-service at http://cmictig.cs.ucl.ac.uk/niftyweb.

### Multi-time-point filling

For the multi-time-point filling, we first calculate the transformation from each time-point to the average position of all time-points using the NiftyReg software package (niftyreg.sf.net). We perform all the pairwise symmetric affine registrations to obtain transformation matrices between each pair of images (Leung et al., 2012). For each image (*i*), we compute the geometric log mean of pairwise affine transformations *T*_*h*_ (Alexa, 2002) as follows:

(3) $$T_{h,i}=\exp\frac{\log\left(T_{i,1}\right)+...\log\left(T_{i,i}\right)...+\log\left(T_{i,N}\right)}{N},\;i\in \left\{1...N\right\}$$

The images and lesion masks are then transformed into an intra-modality/time-point average space. All individual volumes are finally combined into a single 5D volume that contains all available multi-modal and longitudinal information, and inpainted. Note that as an optimal *q* is found for each 5D patch centred at *p*, the same *q* location will be used to fill all the different modalities/time-points at *p*. This allows for a better joint tissue synthesis with more realistic intensities between time points and modalities than if performed individually, thus resulting in more realistic atrophy values. Moreover, the method allows for different lesion masks at each time-point following the normal behaviour of MS lesions that can change with time.

### Parameter settings

All our experiments use the following un-tuned, empirically determined parameters. *T* and *S* patches use an adaptive patch size defined as the maximum Euclidean distance over all time-point images of each *r* lesion voxel to the closest non-lesion voxel *s*. Formally:

(4) $$w{\left(r\right)}_{r\in L}=\underset{\forall i\in I}{\text{argmax}}\left(\mathrm{dist}\left(r,i\right)\right)+1$$

(5) $$\mathrm{dist}\left(r,i\right)=\underset{s\notin L\wedge s\in I^{*}}{\text{argmin}}\left(\sqrt{{\left(r-s\right)}^{2}}\right)$$

The search region Ω is defined as W(*r*) = 4w(*r*). The minimum cardinality of a set of voxels used for computing the patch distance has to be superior to 50%, *i.e. α* = 0.5. Finally, the parameters K and *c* are defined as 0.1 and 2 respectively.

### Using priors

**Fig. 2:**
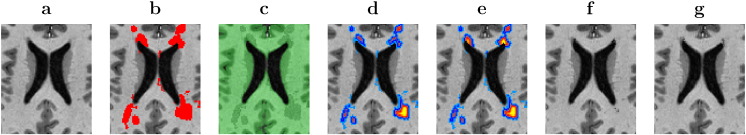
Detail of the ventricles of a healthy subject. From left to right, original image without lesions (*a*). Synthetic lesion mask using the registered lesion mask from the “severe” MS phantom from the *BrainWeb* dataset (*b*). Brain mask obtained using STEPS overlaying synthetic lesion image (*c*). Euclidean distance from each voxel to the closest healthy voxel without defining priors (*d*). Euclidean distance to the closest healthy voxel using as prior mask the STEPS mask (*e*). Filling results without and with priors (*f* - *g*).

Our method can work without any additional priors other than the mask of the regions to be filled. However, we have included an option to add priors to identify valid areas for searching suitable voxels *q* to fill *p* (see Fig. 2). The voxels outside the prior mask are considered as unhealthy voxels and included in the L set of excluded voxels. This extra prior mask is used for defining the adaptive patch size (Eq. (4)) and for getting the voxels that can be used to synthesise the information (Eq. (1)). The extra prior mask is not considered in the computation of the distance between two patches (Eq. (2)).

### Data

To test the performance of the different inpainting methods, three different datasets have been used for different purposes.

The first dataset comes from the public database *BrainWeb* (http://www.bic.mni.mcgill.ca/brainweb/) and it is composed of images with different contrast (T1, T2 and PD) using the “mild”, “medium” and “severe” MS lesion phantom. We will refer to this dataset as BW (BrainWeb).

The second dataset is from 8 healthy, adult volunteers who were recruited for this study to test the algorithm using synthetic lesion generation (age range: 25–45 years). Each subject was scanned 3 times in a month, resulting in a total of 24 scans. This dataset was collected using a 3 Tesla Philips Achieva MRI system (Philips Medical Systems, Best, Netherlands) with an 8-channel head coil resulting in T1-weighted 3D-TFE acquisitions (with an inversion recovery magnetisation preparation) in the sagittal plane with the following imaging parameters: TR = 6.9 ms; TE = 3.1 ms; TI = 867  ms; flip angle *α* = 8; FOV = 256 × 256 mm; voxel size = 1 × 1 × 1 mm; NEX = 1; 180 contiguous slices; scanning time 6 : 30  min. We will refer to this dataset as HV (Healthy Volunteers).

The third dataset is composed of 52 patients with secondary, progressive MS (age range: 30-61 years) and is used for the quantitative analysis. Each subject in this group was scanned at baseline and at 24 months, resulting in a total of 104 scans. After a quality control 11 subjects were discarded due to different image artefacts, resulting in a final dataset of 41 subjects. The MRI data was collected using a single 1.5 Tesla MRI scanner (General Electric, Milwaukee, WI, USA). Quality control and manual lesion segmentation were completed by two trained raters. Appropriate quality assurance procedures, involving regular scanning of control subjects with no known neurological deficit and phantoms, were undertaken in keeping with departmental policy. The following sequences were acquired: 2D T1 W Spin Echo (SE) (TE = 15ms,TR = 550ms, in-plane pixel spacing: 0.9375 × 0.9375 mm, out-of-plane: 3 mm), T2 W Dual Fast SE (TE = 20 ms and 80 ms, TR = 2500ms, voxel size: 0.9375 × 0.9375 × 3  mm) and 3D T1WGE (TE = 5 ms, TR = 15 ms, TI = 450ms, 0.976 × 0.976 × 1.5  mm). This dataset is a subset of placebo group of the Lamotrigine Trial (Kapoor et al., 2010). We will refer to this dataset as MSLA (MS Lamotrigine).

For the second and third datasets, written informed consent was obtained from all participants and the study was approved by our local research ethics committee.

### Synthetic lesion generation

**Fig. 3:**
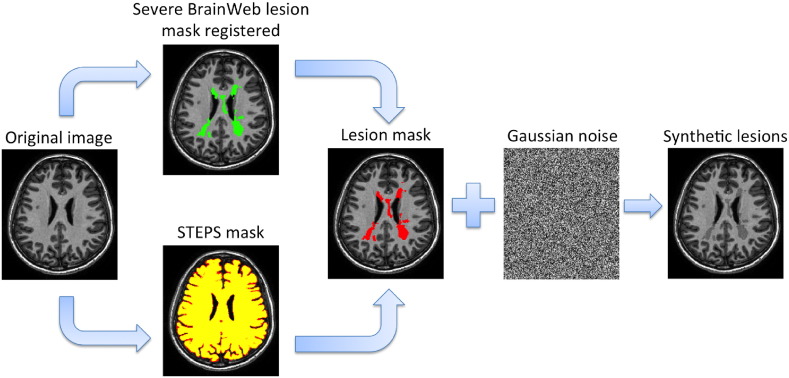
Schema that illustrates the followed main steps to generated the synthetic MS lesions over our healthy subjects.

**Fig. 4:**
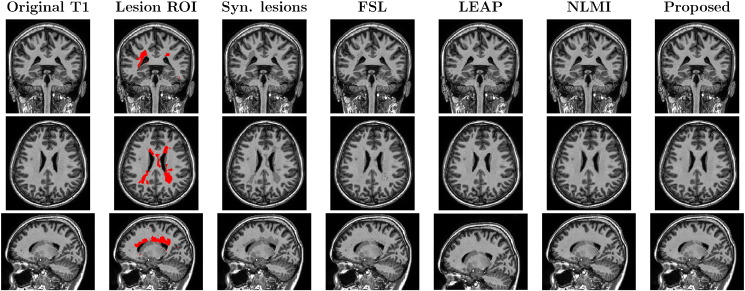
Lesion filling results over a control subject generating synthetic lesions using the registered lesion mask from the “severe” MS phantom from the *BrainWeb* dataset. From left to right, the original T1 image, the lesion mask from *BrainWeb* phantom registered over T1 image after applying the STEPS mask, the generated synthetic lesions, and the fill-lesion results using each method. Each row shows the coronal, axial and sagittal view respectively.

Synthetic lesions were generated and added to the HV data acquired specifically for this study as follows (see Fig. 3, Fig. 4):

• HV brain tissue masks were extracted using STEPS (Cardoso et al., 2013).
• T1 BW images were firstly affine and then non-rigid registered to each scan of the HV using NiftyReg package.
• The binary “severe” lesion mask from BW was resampled to each HV native space.
• The resampled lesion mask was constrained by the STEPS' mask to ensure that all the lesions were inside the brain.
• We generated a normalised Gaussian noise image, which was masked with the resampled lesion mask and multiplied by 0.5, obtaining an intensity profile mask. This is just for a visual propose, because all the inpainting methods used in this paper not consider the values inside the mask.
• Finally, the T1 image of HV was multiplied on a voxel-by-voxel basis by the intensity profile obtained in the previous step to reintroduce spatial variation in the tissue signal intensities and simulate an MS lesion.

### Quantitative analysis' methods

For quantitatively compare different lesion filling approaches, we used 5 measures to assess different aspects of a filling method.

#### Tissue volume errors

We estimated tissue volume errors (Chard et al., 2010) using the mean error (ME) for different segmented-classes. Additionally, we computed the mean absolute error (MAE) to show how close two volumes are. These measures show the influence of each technique over a segmentation method showing how we are changing the tissue classification. We computed the GM, WM and CSF volume using GIF (Cardoso et al., 2012).

(6) $$\mathrm{ME}=100\frac{V_{T}-V_{0}}{V_{0}}$$

where *V*_0_ is the tissue volume before adding synthetic lesions and *V*_*T*_ is the obtained volume after filling the lesions.

#### Tissue homogeneity

We computed the normalised entropy inside the filled lesions. It provides a measure of the tissue homogeneity inside the filled lesions, where small entropy means that all lesions are filled with clustered intensity values.

#### Synthetic tissue integrity

We estimated the gradient magnitude at the edge of the filled lesions |∇* I* |. It provides information on the presence of discontinuities at the boundary of the lesion, with small values meaning a soft transition between real and synthetic intensities and high values show the presence of spurious image gradients at the edge of the region.

#### Synthesisation error

We used the mean square error (MSE) to assess the error in the synthesis process. MSE measures the difference between the synthetic values and the real values within the synthetic lesion regions. Methods, that obtaining a smaller MSE, mean that they fill lesions with more realistic synthetic intensities.

#### Longitudinal filling

We compared the results of estimating longitudinal atrophy with the generalised Boundary Shift Integral (GBSI) pipeline (Prados et al., 2014a, Prados et al., 2014b). GBSI determines atrophy to be localised with areas of intensity changes in the vicinity of the brain boundaries, as determined by the probabilistic segmentations of the aligned baseline and repeat scans (Manjón et al., 2010). To perform the alignment between the two time-points, a symmetric and inverse-consistent registration to the middle space using 12 DOF registration was performed. This technique ensures that findings are not biased due to the registration process. The GBSI intensity window is automatically chosen based on the imaging properties of each of the tissues of interest (Leung et al., 2012). A non-binary XOR region-of-interest is adaptively estimated from probabilistic brain segmentations of both scans, in order to better localise and capture the brain atrophy. Mean and standard deviation of annualised percentage of brain volume change (PBVC) were calculated using each filling method. We estimated sample sizes estimates, along with 95% confidence intervals (CI), for whole brain annualised *PBVC* using a widely accepted formula for these studies (Fox et al., 2000), which provides for 80% power at the 95% significance level to detect a 25% reduction in disease progression compared to the ideal case of 0 atrophy. We obtained bias-corrected bootstrap CIs (10 , 000 samples) for each of the estimated sample sizes.

Statistical analyses were performed using Stata version 10 (College Station, Texas, US).

## Results

Three lesion filling algorithms were used for comparison purposes using T1-weighted images: the lesion automated preprocessing method (Chard et al., 2010), here referred to as LEAP; the filling method implemented in FSL (Battaglini et al., 2012), here referred to as FSL, and the non-local means inpainting method (Guizard et al., 2015), here referred to as NLMI. Public available versions were used for LEAP and FSL. NLMI was reimplemented in-house as the method is not publically available.

### Qualitative analysis

**Fig. 5:**
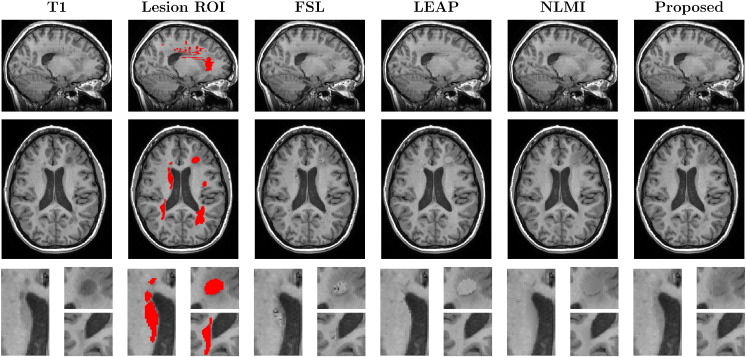
The coronal, axial and zoomed views of the original T1 image of a MS patient and the lesions mask (LROI), followed by the results with FSL, LEAP, NLMI and the proposed methodology. Note the introduction of WM-like intensity in the mis-segmented caudate region (1st zoomed view) using the FSL and LEAP methods.

**Fig. 6:**
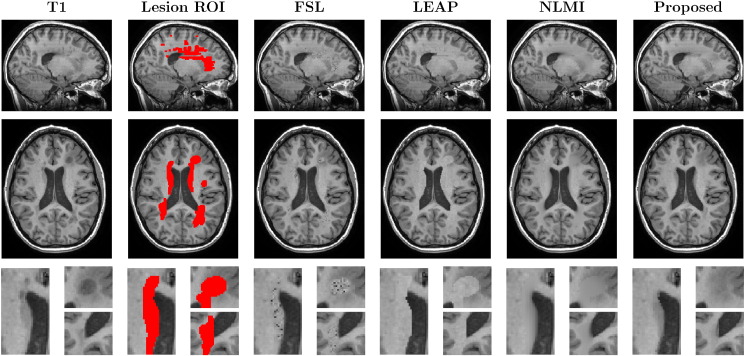
The coronal, axial and zoomed views of the original T1 image of a MS patient and the dilated lesions mask (DROI), followed by the results with FSL, LEAP, NLMI and the proposed methodology. Note the introduction of noisy samples using the FSL method, sharp contrast boundaries with the LEAP method and, the artificial low SNR and blurred boundaries obtained by NLMI method.

In this evaluation, we compare the different methods for filling the lesions of an MS patient in two situations: with L defined as the manually-segmented lesion mask (LROI), and with a dilated version of the same mask (DROI). Fig. 5, Fig. 6 show the results obtained using the original mask and the dilated mask respectively.

**Fig. 7:**
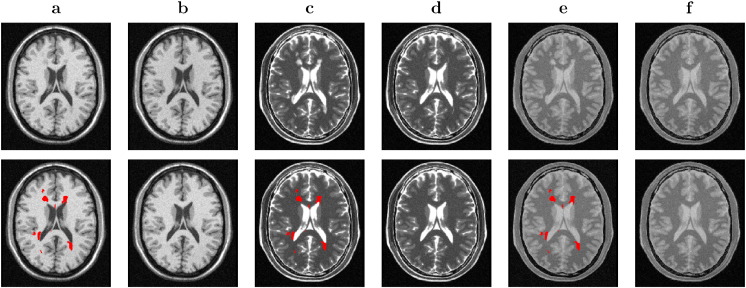
Lesion filling results on different modalities using the “severe” MS phantom from the *BrainWeb* dataset. First row, the T1 and the lesion filled T1 (*a* - *b*), the T2 and the lesion filled T2 (*c* - *d*), and the PD and the lesion filled PD (*e* - *f*). Second row, the lesions mask over each different modality image (a, c and e), and the ground truth for each different modality image (b, d and f).

Furthermore, the proposed method was also applied to images from different modalities from BW dataset (see Fig. 7), demonstrating its immediate generalisation and agnosticism to the type of image contrast. The same parameters were used for lesion filling all modalities.

### Quantitative analysis

In this section, we quantitatively assessed which method produced the most realistic patch. In order to do so, we applied our synthetic lesion generation method over our HV dataset. We computed the normalised entropy inside the filled lesions and the gradient magnitude at the edge of the filled lesions (|∇* I* |). We used the mean square error (MSE) to measure the difference between the synthetic values and the real values within the ROI region, with a smaller MSE meaning more realistic synthetic intensities in the filled ROI. Also, we computed the different tissue volume changes. All results are presented in Table 1.

Furthermore, we tested the different methods over MSLA dataset. We filled all the images and computed the atrophy between time-points using GBSI. Table 2, Table 3 show the results obtained in terms of atrophy and sample size respectively.

## Discussion

Our algorithm has been tested in three different scenarios: public database, healthy dataset and MS dataset.

In the first scenario, we have evaluated visually the quality of the different methods. Fig. 5, Fig. 6 show that the proposed method not only preserves better the boundaries of the underlying neighbouring structures (ventricles and WM/GM boundaries), but also reduces artefacts and spurious rasterisation due to lesion shape, size and position and due to the choice of imaging plane for manual segmentation. Furthermore, as the proposed method is context aware, it is also able to cope with situations when the human rater erroneously segments a non-pathological region of interest, *e.g.* the first zoomed region in both Fig. 5, Fig. 6 shows that the caudate nucleus was mislabelled as an MS lesion. This structure was correctly preserved using the proposed technique but not using both the LEAP and FSL techniques.

In Fig. 7, the generalisability and agnosticism to the type of tissue contrast is demonstrated. T1-, T2- and PD-weighted images are filled successfully using the same parameters for all of them.

The quantitative evaluation was performed simulating lesions over a healthy dataset. The results demonstrate that the proposed method using priors fills the lesions with the most similar intensity to the previous one. The obtained entropy, gradient and MSE values show that our method is generating the most similar filling intensities to the expected normal tissue with statistically significant difference respect the other methods (see Table 1). The FSL method obtains slightly better (lower) gradient values across the lesion boundaries due to the fact that it fills the lesion boundary with samples taken from the surrounding histogram. The lower entropy obtained by LEAP is because it fills all the lesions with the same intensity (mean WM intensity) adding a small Gaussian error, resulting in the fact that the synthetic values are close to each other having a smaller variability.

ME and MAE values show that FSL and NLMI methods tend to expand the GM volumes. LEAP tends to reduce CSF volume whilst NLMI expands it. The proposed method without priors tends to generate synthetic CSF-like intensities in overly large periventricular lesions. These biases at tissue boundaries affect the atrophy estimates as computed using GBSI.

In terms of tissue classification LEAP fills the lesions using class-specific intensities, with the consequence of losing CSF volume whilst increasing the GM and WM volumes. This is caused by lesions that are close to the ventricle boundaries that are classified as WM or GM rater than CSF, after filling them using LEAP. This results in an atrophy overestimation.

The new methods ME and MAE values show that it tends to slightly overestimate CSF tissue, although this bias is less than with the other techniques. This is happening at the tissue-ventricles interface and could result in a fictitious change of ventricles boundaries at different time-points that means an atrophy overestimation. However, after visual inspection, we have seen that this happens with the bigger periventricular lesion loads. The proposed solution to this problem, *i.e.* the introduction of priors and the longitudinal filling, helps to fill the lesions appropriately with minimum spurious synthetic CSF.

The longitudinal analysis, that we performed using the MS dataset, demonstrates the gains obtained by filling lesions longitudinally by taking into account the temporal evolution of lesions. The smaller sample size required by our longitudinal inpainting method (see Table 3) shows that it is better to apply a longitudinal image synthesis method than filling each time-point separately, simulating the real tissue changes between time-points. New and changing lesions are filled more consistently thanks to the additional information provided by the other time points where the tissue might not have been corrupted. Nonetheless, differences between the longitudinal and non-longitudinal methods were not found to be significant in our study, possibly due to the small test dataset (*N* = 41). However, it should be noted that filling each time-point separately, as in the non-longitudinal approach, can generate synthetic atrophy if the patches selected in different time-points to fill the same lesion are sourced from different brain areas. This can lead to darker intensity patches being used in some time-points *versus* lighter ones in others, producing intensity variations that can resemble atrophy. This limitation is avoided in the longitudinal approach.

Furthermore, the proposed method is not only less affected by the lack of contrast between tissues, as it fills the lesion ROI with the most similar non-local patches and not according to a class-specific intensity model, but at the same time more robust to the location of the lesions, *i.e.* previous algorithms have problems with lesions located close to non white matter regions. Filling lesions with intensity values from the surrounding areas could be a problem from histological point-of-view, but not from image synthesis point-of-view, since the synthesis process makes that the affected area have values similar to the expected look like and the final aim is that all the posterior image processing methods get the expected results, as we were using healthy brains.

The NL-Means models typically use a weighted average of the best-matched patches, rather than using the best match itself. In our testing, we found that weighted averaging can introduce blurring and an artificially low SNR in the filled areas (see Fig. 4, Fig. 5, Fig. 6 for NLMI *vs.* Proposed method), making it more difficult to estimate longitudinal tissue differences as demonstrated in Table 2, Table 3.

Lastly, manual lesion editing is still the gold standard for lesion masking in MS, with the accuracy of the rater and the choice of segmentation plane being sources of bias. By exploiting contextual information, the proposed algorithm has been shown to be more robust to lesion over-segmentation than previously published techniques. Thus, it would be interesting to see if the proposed method can be used in conjunction with a highly sensitive automatic lesion detection methodology, thus removing rater bias from the analysis process.

Finally, although the NLMI method (Guizard et al., 2015) also uses an NL-means strategy similar to the proposed method, the two algorithms are significantly different. The NLMI method always uses the whole patch, whilst the method presented here uses only non-lesion voxels within a size-adaptive patch. The concentric filling strategy of the proposed method also allows for previously inpainted voxels to contribute towards the patch distance of inner voxels. Conversely, rather than filling lesions concentrically, NLMI fills the image hierarchically at different resolutions and with different smoothing factors to enable the propagation of intensities over longer distances. Finally, NLMI inpaints multiple time points independently whilst the proposed method does so jointly.

## Conclusion

In this paper, we propose a new and robust multi-modality and multi-time-point lesion filling technique that relies on a non-local patch match strategy. The method shows improved results compared to previously published publicly available methods. We have demonstrated that the presented method is able to fill the lesions with the most plausible values in different tissue contrast and in multi-time-points at the same time. Future work will explore a multi-subject patch search technique and a model parameter optimisation.

## Figures and Tables

**Table 1:** Results from the quantitative analysis, with the mean (std) and a * when the pair *t*-test (respect to proposed method using priors) got *p* < 0.05 over the 24 images for the healthy subjects group. First row is the lesion region entropy after filling. Second row is the gradient magnitude |∇* I* | respect the neighbourhood. The third row shows the mean square error (MSE) in comparison with the real tissue value. Rows 4–6 show the mean error (ME), the paired *t*-tests are between the obtained volumes for each tissue respect to the obtained by the proposed method using priors. Finally, rows 7–9 show the mean absolute error (MAE) for each tissue segmentation.

	FSL	LEAP	NLMI	Proposed	Prop. with priors
Entropy	0.200 (0.027)*	0.098 (0.015)*	0.141 (0.022)*	0.165 (0.026)	0.164 (0.026)
|∇I ∥	0.040 (0.009)*	0.060 (0.012)*	0.045 (0.009)*	0.042 (0.009)*	0.043 (0.009)
MSE	0.146 (0.085)*	0.403 (0.297)*	0.011 (0.011)*	0.021 (0.020)*	0.006 (0.005)
ME(GM)	0.516 (0.149)*	0.127 (0.076)*	1.064 (0.445)*	-0.057 (0.049)*	-0.035 (0.061)
ME(WM)	-0.392 (0.098)*	0.052 (0.072)*	-0.146 (0.244) *	0.015 (0.056)	0.010 (0.059)
ME(CSF)	0.055 (0.171)	-0.395 (0.261)*	0.792 (0.365)*	0.056 (0.162)*	0.005 (0.183)
MAE(GM)	0.516 (0.149)*	0.130 (0.071)*	1.064 (0.445)*	0.064 (0.039)	0.057 (0.039)
MAE(WM)	0.392 (0.098)*	0.075 (0.045)*	0.200 (0.199)*	0.046 (0.034)	0.044 (0.040)
MAE(CSF)	0.153 (0.089)	0.395 (0.261)*	0.792 (0.365)	0.132 (0.107)	0.147 (0.105)

**Table 2:** First row shows the mean and standard deviation of the whole brain PBVC. Below, a comparison between methods is done, the difference in mean with the 95% CI and paired *t*-test is shown.

*N* = 41	FSL	LEAP	NLMI	Prop. non-Longitudinal	Prop. Longitudinal
Mean (Std.)	0.956 (0.540)	0.910 (0.514)	0.928 (0.540)	0.921 (0.509)	0.934 (0.512)



Difference in mean (95% CI), *p*-value	
FSL *vs* LEAP	0.045 (-0.002 to 0.093), *p* = 0.06
FSL *vs* NLMI	0.028 (-0.015 to 0.071), *p* = 0.19
FSL *vs* Prop. non-Longitudinal	0.035 (0.005 to 0.065), *p* = 0.06
LEAP *vs* NLMI	-0.017 (-0.059 to 0.025), *p* = 0.42
LEAP *vs* Prop. non-Longitudinal	-0.010 (-0.053 to 0.032), *p* = 0.62
NLMI *vs* Prop. non-Longitudinal	0.007 (-0.044 to 0.031), *p* = 0.72
FSL *vs* Prop. Longitudinal	0.021 (-0.049 to 0.092), *p* = 0.54
LEAP *vs* Prop. Longitudinal	-0.024 (-0.073 to 0.025), *p* = 0.33
NMLI *vs* Prop. Longitudinal	-0.007 (-0.070 to 0.056), *p* = 0.82
P. non-Longitudinal *vs* Prop. Longitudinal	-0.013 (-0.081 to 0.054), *p* = 0.69

**Table 3:** Estimated sample sizes (95% CI) in comparison to the ideal case of no-atrophy using whole brain annualised PBVC (80% power at the 5% significance level to detect 25% reduction in disease progression). Below, it is shown the percentage difference of sample size with the 95% CI and paired *t*-test between methods.

*N* = 41	FSL	LEAP	NLMI	Prop. non-Longitudinal	Prop. Longitudinal
Sample size	80 (52 to 125)	80 (50 to 129)	85 (54 to 134)	77 (51 to 117)	75 (46 to 125)



Percentage difference in mean (95% CI), *p*-value	
FSL *vs* LEAP	-0.2 (-22.7 to 22.2), *p* = 0.98
FSL *vs* NLMI	6.3 (-14.0 to 26.8), *p* = 0.53
FSL *vs* Prop. non-Longitudinal	-4.0 (-15.9 to 7.9), *p* = 0.51
LEAP *vs* NLMI	6.6 (-12.0 to 25.3), *p* = 0.48
LEAP *vs* Prop. non-Longitudinal	-3.8 (-22.7 to 15.1), *p* = 0.69
NLMI *vs* Prop. non-Longitudinal	-9.8 (-26.0 to 6.4), *p* = 0.23
FSL *vs* Prop. Longitudinal	-5.6 (-38.4 to 27.0), *p* = 0.74
LEAP *vs* Prop. Longitudinal	-5.4 (-31.8 to 21.9), *p* = 0.69
NMLI *vs* Prop. Longitudinal	-11.3 (-34.8 to 12.2), *p* = 0.34
P. non-Longitudinal *vs* Prop. Longitudinal	-5.9 (-33.6 to 21.7), *p* = 0.67
